# Blockade of Kv1.3 Potassium Channels Inhibits Differentiation and Granzyme B Secretion of Human CD8+ T Effector Memory Lymphocytes

**DOI:** 10.1371/journal.pone.0054267

**Published:** 2013-01-30

**Authors:** Lina Hu, Tongguang Wang, Anne R. Gocke, Avindra Nath, Hao Zhang, Joseph B. Margolick, Katharine A. Whartenby, Peter A. Calabresi

**Affiliations:** 1 Department of Neurology, Johns Hopkins University School of Medicine, Baltimore, Maryland, United States of America; 2 Section of Infections of the Nervous System, National Institute of Neurological Disorders and Stroke, National Institutes of Health, Bethesda, Maryland, United States of America; 3 Department of Molecular Microbiology and Immunology, Bloomberg School of Public Health, Johns Hopkins University, Baltimore, Maryland, United States of America; Shanghai Jiao Tong University School of Medicine, China

## Abstract

Increased expression of the voltage-gated potassium channel Kν1.3 on activated effector memory T cells (T_EM_) is associated with pathology in multiple sclerosis (MS). To date, most studies of Kν1.3 channels in MS have focused on CD4+ T_EM_ cells. Much less is known about the functional relevance of Kv1.3 on CD8+ T_EM_ cells. Herein, we examined the effects of Kν1.3 blockade on CD8+ T cell proliferation, differentiation into cytotoxic effector cells, and release of granzyme B (GrB), a key effector of CD8+ T cell-mediated cytotoxicity. We confirmed the expression of Kv1.3 channels on activated human CD8+ T lymphocytes by immunofluorescent staining. To test the functional relevance of the Kv1.3 channel in CD8+ T cells, we inhibited this channel via pharmacological blockers or a lentiviral-dominant negative (Kv1.xDN) approach and determined the effects of the blockade on critical pathogenic parameters of CD8+ T cells. We found that blockade of Kv1.3 with both lentivirus and pharmacologic agents effectively inhibited cytotoxic effector memory cells’ proliferation, secretion of GrB, and their ability to kill neural progenitor cells. Intriguingly, the KvDN transduced T cells exhibited arrested differentiation from central memory (T_CM_) to effector memory (T_EM_) states. Transduction of cells that had already differentiated into T_EM_ with KvDN led to their conversion into T_CM_. CD8+ T_EM_ have a critical role in MS and other autoimmune diseases. Our present results indicate a critical role for Kv1.3 in the conversion of CD8+ T cells into potential pathogenic effector cells with cytotoxic function.

## Introduction

T cell activation and proliferation rely on the sustained influx of calcium through the calcium release activated calcium (CRAC) channels for efficient signal transduction and gene transcription. Two types of K^+^ channels, termed voltage-gated Kν1.3 and Ca^2+^-activated KCa3.1 (also known as IKCa1), contribute to the control of calcium signaling by providing the electrical driving force for calcium entry. Kν1.3 channels are activated by depolarization of the membrane and restore the resting membrane potential of T cells by an outward flux of potassium ions [1]. KCa3.1 channels, on the other hand, are initially activated by the rise of the cytosolic free calcium concentration and consequently hyperpolarize the membrane, resulting in enhanced calcium influx [1]. The operation of K^+^ channels controlling calcium signaling depends on which channel dominates in the cells.

There are four distinct CD8+ T cell subsets, naïve (CD45RA+CCR7+), central memory (T_CM_, CD45RA−CCR7+) and effector memory (T_EM_) subsets composed of CD45RA-T_EM_ (T_EM_, CD45RA−CCR7−) and CD45RA+ T_EM_ (T_EMRA_, CD45RA+CCR7−), which display differential expression of Kν1.3 and KCa3.1 channels depending on their state of activation and differentiation. In the absence of substantial stimulatory signals, all CD8+ T cell subsets in their quiescent state express a comparable number of Kν1.3 and KCa3.1 channels, with Kv1.3 channels five- to fifty-fold more abundant than KCa3.1 [1], [2], [3], [4]. Upon activation and subsequent differentiation of naïve and T_CM_ CD8+ T cells into effector cells, KCa3.1 channels are rapidly and significantly augmented relative to Kν1.3, thus acting as the predominant functional K+ channel in activated naïve and T_CM_ subsets. In contrast, activation of T_EM_ CD8+ T cells substantially increases the numbers of Kν1.3 channels, but does not significantly change the levels of KCa3.1; thus, the numbers of active K+ channels may be a specific functional marker that could distinguish effector capacities. Since Kv1.3 channels are functionally dominant in activated T_EM_ T cells, while KCa3.1 channels are dominant in activated naïve and T_CM_ T cells it is possible to target these subsets differentially by administration of specific K+ channel blockers [1], [2], [3], [4].

While MS has traditionally been thought to be mediated by CD4^+^ T cells, increasing evidence suggests that infiltrating CD8^+^ T cells may be a key factor in mediating CNS damage. For example, infiltrating CD8^+^ cells were found to be 10-fold more abundant than CD4^+^ T cells in MS brain lesions [5], while expanded CD8^+^ T cells persist in the CSF [6] and dominate the T-cell infiltrate in MS brain tissue [7], [8]. Adoptive transfer of CD8^+^ T cells reactive to MBP [9], [10] or MOG [11] was found to be capable of inducing severe EAE in animal models, and CD8^+^ T cell lines recognizing MBP derived from MS patients were able to cause injury to oligodendrocytes [12]. In addition, persistently expanded CD8^+^ T cells present at high density in the CSF of MS patients express a memory phenotype, and the CD8^+^ cytotoxic T cell response to MBP is increased in MS patients compared to healthy controls [8], [12].

The signals that lead to the development of pathogenic CD8^+^ T cells are not well understood. Our previous findings showed that the infiltrating CD8^+^ cells with effector memory phenotype in MS brain tissue expressed high levels of the Kν1.3 channel [13]. We thus hypothesize that the activated autoreactive T_EM_ of both the CD4^+^ and CD8^+^ T cells expressing a Kν1.3^high^ phenotype may play an important pathogenic role in MS, possibly by exhibiting cytotoxic functions responsible for axonal damage [14].

One likely mechanism by which both CD4^+^ and more so CD8^+^ T cells induce damage in MS is through the release of granzyme B (GrB). GrB-expressing CTLs within actively demyelinating lesions were frequently found in close proximity to injured axons [14]. Activated CD8^+^ T cells were shown to induce neuronal toxicity by releasing GrB and this was reversed by specific GrB immundepletion [15], [16], indicating that release of this molecule may be important mechanism of neurotoxicity in MS. It is important to note that since the autoantigen(s) in MS remain unknown and are likely heterogenous between and even within individual patients, we chose to activate T_EM_ CD8^+^ T cells derived from healthy controls by crosslinking TCR rather than introducing a bias by picking specific peptides and types of patients. Our studies provide proof of concept that signaling through the Kv1.3 channel is a novel mechanism by which GrB is released and suggest an additional mechanism by which blockade of this channel might be therapeutic in MS and other diseases in which cytoxic T cells damage their target tissue.

## Materials and Methods

### Cell Isolation and Stimulation

Human peripheral blood mononuclear cells (PBMC) were isolated from whole blood obtained from 13 healthy volunteer donors via venipuncture (30 mL total volume), followed by differential density gradient centrifugation as described previously [17]. The study was in accordance with the Declaration of Helinski, approval was obtained from the Johns Hopkins University Institutional Review Board and all participants gave their written informed consent.

CD8 subsets were obtained by negative selection using magnetic microbeads (Miltenyi Biotec, Auburn, CA) according to the manufacturer’s instructions. The purity of human CD8+ T cells was consistently >95% as routinely checked by FACS analysis. Purified CD8+ T cells were adjusted to 1 to 2×106/ml in T cell medium [Iscoves’s modified Dulbecco’s Medium (IMDM) supplemented with glutamine (2 mM), penicillin (100 U/ml), streptomycin (100 µg/ml), gentamicin (50 µg/ml) (Biowhittaker, East Rotherford, NJ) and 5% human serum (Sigma, St. Louis, MO)]. The cells were stimulated with anti-CD3 alone (cells:beads, 1∶1) or anti-CD3/CD28 (cells:beads, 10∶1) mAb-conjugated magnetic beads (Dynal Biotech, Brown Deer, WI). The higher ratio of cells to beads for anti-CD3 alone as compared to anti-CD3 and anti-CD28 was necessary to obtain measurable cell activation, proliferation and granzyme B secretion.

### Pharmacologic Channel Blockers

Four K^+^ channel blockers were used at their IC50s in the functional assays including: *Stichodactyla helianthus* toxin (ShK), a potent Kv1.3 inhibitor [18]; Margatoxin (MgTx), a specific Kv1.3 inhibitor [19], [20]; Charybdotoxin (ChTx), which inhibits both Kv1.3 [21], [22], [23] and KCa3.1 [24], [25] channels with similar potency; and 1-[(2-chlorophenyl)diphenylmethyl]-1*H*-pyrazole (TRAM-34), which selectively blocks the KCa3.1 channel [26]. Charybdotoxin (ChTx) and margatoxin (MgTx) were purchased from Alomone Labs (Jerusalem, Israel). ShK and TRAM-34 were kindly provided by Dr. Michael Pennington (Bachem) and Dr. Wulff Heike (University of California), respectively.

### Immunofluorescence Microscopy

Cells were washed and placed into cytospin funnels and spun onto glass slides using a cytospin centrifuge (Shandon, Pittsburgh, PA) at 750 rpm for 5 minutes. Cells were subsequently fixed with 3.7% paraformaldehyde for 15 min. For intracellular staining, cells were fixed, permeabilized with Cytofix/Cytoperm solution (PharMingen, San Diego, CA). After thorough washing of cells, non-specific binding sites were blocked using 0.5% BSA in PBS for 15 min at room temperature. Thereafter, cells were incubated with rabbit anti-human Kv1.3 (Alomone Labs, Jerusalem, Israel), mouse anti-human GrB (Caltag Laboratories, San Francisco, CA) or mouse anti-human CD8 (PharMingen) antibodies for 30 min at room temperature. Cells were then labeled with appropriate secondary antibodies conjugated to Alexa Fluor (AF)-488 and AF-594 (Molecular Probes, Eugene, OR). Cellular nuclei were stained with 4, 6-diamidino-2-phenylindole (DAPI) (Molecular Probes) at 1 µg/ml for 10 min. After being mounted in Immuno Fluore medium (ICN Biomedicals, Aurora, OH), images were acquired by OpenLab software on a Zeiss Axiovert S100 microscope under 100X objective (Carl Zeiss, Thornwood, NY).

### Flow Cytometric Analysis and Cell Sorting

Single cell suspensions were prepared and stained as previously described [2], [17]. The monoclonal Abs utilized for the staining were FITC-anti-CD45RA (PharMingen), PE-anti-CCR7 (PharMingen), PerCP-anti-CD8 (PharMingen), Cy-Chrome-anti-CD8 (R & D systems), APC-anti-CD45RA (PharMingen), FITC-anti-CD45RA (PharMingen) and APC-anti-GrB (GB12; Caltag, Burlingame, CA) or appropriate isotype controls. Briefly, cells were washed twice in PBS/0.5% BSA and incubated with a mixture of specific Abs against surface molecules for 30 min on ice. Cells were washed twice again in PBS/0.5% BSA, and fixed, permeabilized with Cytofix/Cytoperm solution (PharMingen). Subsequently, cells were incubated with APC-anti-GrB Ab or appropriate isotype control and diluted at 1∶200 for 30 min on ice, washed twice in PBS/0.5% BSA and then analyzed on a FACSCalibur flow cytometer using CellQuest software (BD Immunocytometry Systems, San Jose, CA).

The CD8+ cells were separated into naive, T_CM_, T_EM_ and T_EMRA_ subsets, by cell sorting using the combination of anti-CD8-Cy-Chrome, anti-CCR7-PE and anti-CD45RA-FITC mAbs. Single cell suspensions were stained, and the naive, T_CM_, T_EM_ and T_EMRA_ cells within the gate of CD8+ cell population were sorted based on their differential expression of CCR7 and CD45RA using a MoFlo MLS high-speed cell sorter (Dako, Fort Collins, Colorado). The purity of each sorted population was consistently >95%.

### [^3^H] Thymidine Incorporation

Freshly isolated CD8+ T cells were pretreated with channel blockers for 3 h. The cells were then cultured at 2×105 cells/well in triplicate in 96-well flat-bottom plates (Falcon, Franklin Lake, NJ) in 0.2 ml IMDM/5% human serum/1× penicillin-streptomycin under the stimulation conditions as described above for 4 to 5 days. Cultures were pulsed with 1 µCi of [3H] thymidine (Amersham Biosciences, Piscataway, NJ) for the final 18 h. Cells were harvested by a 96-well plate harvester (PerkinElmer, Turku, Finland), and [3H] thymidine uptake was measured in a liquid scintillation counter (PerkinElmer).

### Lentiviral Transduction of Activated CD8+ T cells

T cells were transduced with a lentivirus construct containing the GFP reporter gene and a dominant negative (DN) form of the Kv1.x channel as previously described [27]. The DN Kv1.x sequence codes for a Kv1.x molecule with a function-blocking mutation (GYG to AYA) in the pore-forming region, which prevents formation of the tetrameric complex in the membrane [28], [29]. Its denotion of Kv1.x indicates it affects the Kv1 family, but since human T cells are only known to express Kv1.3, this channel is likely to be the only one affected.

To obtain activated CD8+ T cells, highly purified CD8+ and sorted CD8 subsets were stimulated with anti-CD3/CD28 for 24 h. IL-2 was not added to avoid it’s known effects on bypassing Kv1.3 signaling. Lentiviral transduction of activated CD8 cells was performed in 48-well plates containing 2 ml/well of T cell culture medium in the presence of 8 µg/ml protamine sulfate (Sigma); cells were mixed with Lenti-GFP or Lenti-DN Kv1.x vector particles at an MOI of ∼5, centrifuged at 2000 r.p.m. for 30 min at 32°C, and incubated at 37°C. T-cell cultures were maintained by restimulating every 10 days with anti-CD3/CD28 Abs for 3 weeks. Transduction efficiency was determined at different time points (days 3, 7, 14 and 21 following infection) by examining GFP expression by flow cytometry. Non-infected T-cells, cultured under the same conditions, were used as a negative control for GFP. The transduction efficiencies were usually ∼30% and less than 10% of the cells died during transduction. We did not observe any preferential transduction of CD8 cell subsets.

### PKH26 Staining

Transduced CD8 T cells were suspended in PBS containing 1% FCS at a concentration of 10^7^cells/ml and labeled with the fluorochrome PKH26 (SIGMA). Cells were incubated at 25°C for 5 min with PKH26 at a final concentration of 2 µM, washed twice with, and then resuspended in IMDM/5% human serum/1× penicillin-streptomycin. Division of cells was measured via dilution of PKH26 using flow-cytometric analysis after 5 days of culture under the stimulation conditions as described above.

### GrB ELISA Analysis

Supernatants were collected from cultures and were then frozen at −80°C. The frozen supernatants were thawed at room temperature and GrB levels were measured with commercial ELISA assay kit (Cell Sciences, Canton, MA) according to the manufacturers' instructions.

### Neurotoxicity Assays

Human neural progenitor cells (NPCs) were cultured from human fetal brain specimens of 7–8 weeks of gestation obtained from Birth Defects Research Laboratory, University of Washington, Seattle in accordance with National Institutes of Health (NIH) guidelines and following approval by the Institutional Review Board at the Johns Hopkins University and NIH as described previously [15], [16]. NPC were cultured at 1×105/ml in neural differentiation media (DMEM/F12 containing 2% FBS) for neuronal induction in 96-well plates for seven days and then were exposed for an additional 24 hours to conditioned media (1∶20 to dilution) from 72 hour activated CD8+ T cells treated with MgTx or vehicle control. Neurotoxicity was evaluated by using CellQuanti-BlueTM Cell Viability Assay Kit (BioAssay Systems, Hayward, CA). CellQuanti-blue solution (10 ul/well) was added to the cultures and cells were incubated for 30 min. The fluorescence intensity was then detected at Excitation 530 nm and Emission 590 nm.

### Statistical Analysis

Statistical evaluation of significance between the experimental groups was determined by Student’s *t* test. Results were determined to be statistically significant when *p*≤0.05.

## Results

### K_v_1.3 is Upregulated in Activated CD8+ T cells

**Figure 1:**
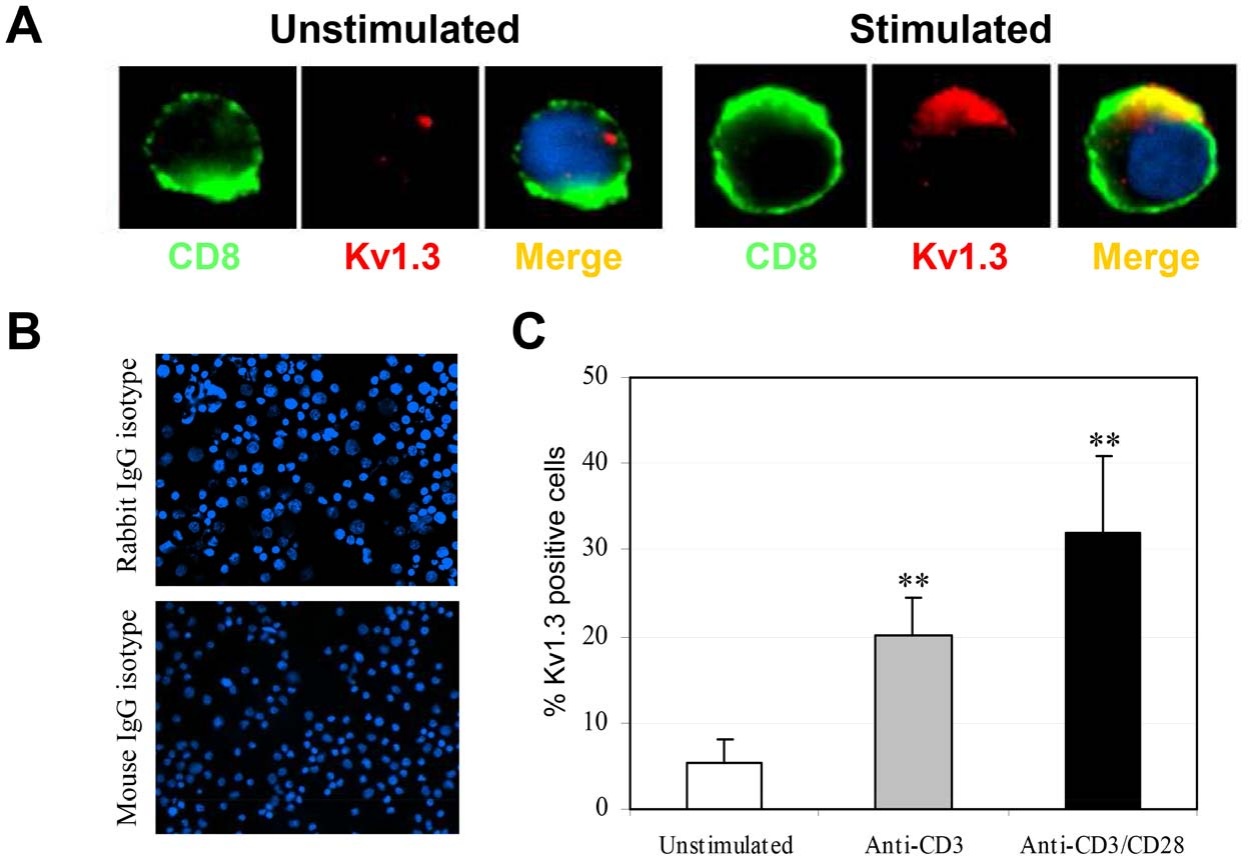
Kv1.3 expression is upregulated in activated CD8+ T cells and co-localizes with CD8. (A) Purified CD8+ T cells were stimulated with anti-CD3/CD28 for 3 days. Naïve and stimulated cells were then immunostained for Kv1.3 in combination with CD8 and subsequently viewed by immunofluorescence microscopy. Cellular nuclei were counterstained with DNA dye DAPI (blue). Kv1.3 detected by AF 594 fluorescence is shown in red, while CD8 detected by AF 488 fluorescence is shown in green. Colocalization is indicated by a yellow and/or orange color in the overlay panels. (B) An isotype-matched antibody was used as a negative control. Original magnification, ×100. Image is representative of three different donors. (C) Summary of percentages of activated CD8+ T cells expressing Kv1.3. In brief, 4 view fields/microscopic section were evaluated for Kv1.3+ CD8 cells stimulated with anti-CD3/CD28 or anti-CD3 alone for 3 days. The percentages of Kv1.3+ cells are based on the number of CD8+ T cells counted. Data are mean ± SD from one representative of three independent and reproducible experiments. Values that are significantly different from that of non-stimulated control are indicated as **, *p*<0.01.

To quantify the CD8^+^ T cells expressing K_v_1.3 at rest and after stimulation, freshly purified CD8^+^ T cells (approximately 60% T_EM_/T_EMRA_) were either left unstimulated or stimulated with anti-CD3 and anti-CD28 antibodies for 3 days, then immunostained for K_v_1.3 and CD8 and analyzed by fluorescence microscopy. Images shown in Fig. 1A demonstrate that while resting CD8^+^ T cells failed to express detectable amounts of K_v_1.3, activation of these T cells with anti-CD3/CD28 resulted in a strong upregulation of K_v_1.3 in the cell membrane, which was colocalized with the surface CD8 domain. Similar results were also obtained from anti-CD3 stimulated CD8^+^ T cells (data not shown). The frequency of CD8^+^ T cells expressing membrane K_v_1.3 was significantly increased after stimulation with anti-CD3/CD28 and anti-CD3 alone (Fig. 1 B and C).

### Kv1.3 Blockade Preferentially Suppresses the Proliferation and Differentiation of T_EM_ Cells

**Figure 2:**
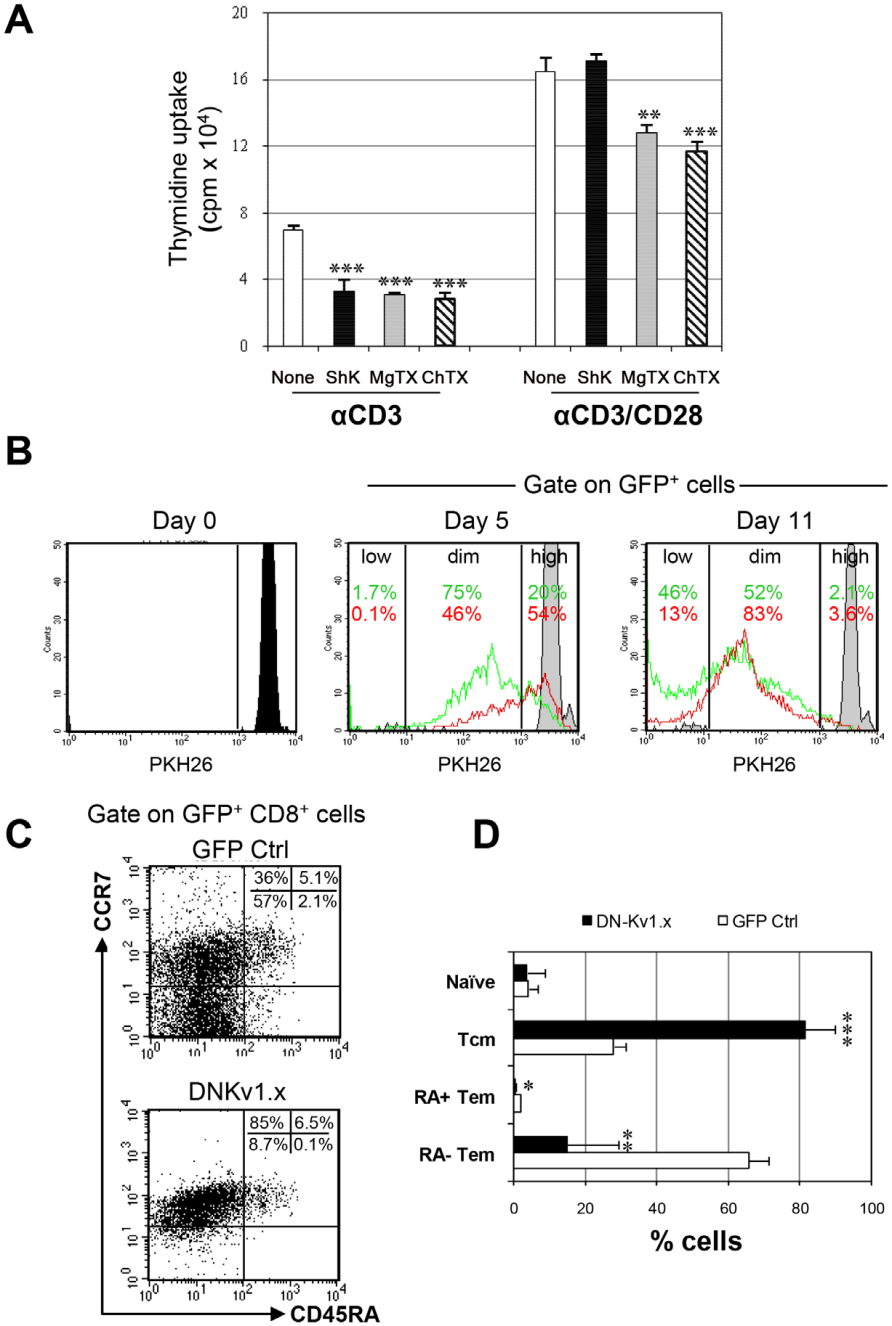
Kv1.3 blockade suppresses proliferation and differentiation of anti-CD3 stimulated CD8+ T cells. *(* A) Freshly isolated CD8+ T cells were pretreated with Kv channel blockers, ShK (10 nM), MgTx (30 nM) and ChTx (50 nM), for 3 hours, then stimulated with anti-CD3 alone or anti-CD3/CD28. After 4 days of culture, proliferation was measured by [3H] thymidine uptake. Data show the mean ± SD of three experiments. Significant differences are marked as follows: (*, *p*<0.05; **, *p*<0.01; ***, *p*<0.005). (B). Isolated CD8+ T cells were labeled with PKH26 stimulated with anti-CD3/CD28 for 24 h, and then transduced with a lentiviral vector encoding the dominant-negative Kv1.x or the GFP control alone. PKH26 fluorescence was analyzed by flow cytometry at baseline and 5 and 11 days later as shown. Quantification of proliferating cells was evaluated by gating on PKH26high PKH26dim and PKH26low among GFP+ cells. (C). Transduced CD8+ T cells were stained with anti-CD8, anti-CCR7 or anti-CD45RA mAbs seven days after transduction and analyzed for the percentages of naïve, T_CM_, T_EMRA_ and T_EM_ cells in gated GFP+ CD8+ cells. FACS plots shown are representative data from three separate experiments using cells from different donors. (D) The percentages of each CD8+ subset displaying GFP fluorescence are presented as mean ± SD of three experiments. Values that are significantly different from that of GFP control are indicated as follows: **, *p*<0.01; ***, *p*<0.005.

We employed two distinct approaches to assess the functional relevance of Kv1.3 expression by activated CD8^+^ cells. First, we assessed the role of Kv1.3 in proliferation of CD8^+^ T cells by blocking Kv1.3 signaling with pharmacologic agents. Purified CD8^+^ T cells were stimulated in a dose response (data not shown) of anti-CD3 or anti-CD3/CD28 in the presence or absence of blockers: ShK and MgTx (Kv1.3 blockers) or ChTx (Kv1.3 and KCa3.1 blocker). Cell proliferation of the CD8^+^ cells was measured by [^3^H] thymidine incorporation four days later. As shown in Fig. 2A, proliferation of CD8 T cells treated with ShK was markedly reduced compared with that of non-treated cells when the cells were stimulated with anti-CD3, whereas there were no significant differences in responses to anti-CD3/CD28 of CD8^+^ cells between ShK- and non-treated cells. Treatment of CD8^+^ T cells with ChTx and MgTx inhibited cell proliferation induced by both anti-CD3/CD28 and anti-CD3 alone. Since the pharmacological K^+^ blockers are rapidly degraded, which limits evaluation of sustained channel blockade, we sought to utilize an alternate approach to assess the durable effects of channel blockade on cell proliferation and differentiation. To this end, purified PKH-labeled CD8^+^ T cells were transduced with a GFP-tagged, lentiviral vector expressing a DN Kv1.x sequence or GFP alone as a control at saturating concentration (MOI) of LV without inducing cell death. The proliferative response of these transduced CD8^+^ cells was subsequently examined by the PKH26 dilution five and eleven days later after TCR cross-linking. As shown in Figure 2B, the modification of cells with DNKv decreased the numbers of divided cells, relative to control modified cells, at both timepoints. We also analyzed the distribution of subpopulations among GFP^+^CD8^+^ cells seven days after transduction. Of note, transduction of cells with DNKv led to a significant decrease in the number of T_EM_ CD8^+^ cells and a corresponding increase in T_CM_ cells, relative to the GFP control (Fig. 2C and D). However, within the naïve subset, no significant differences were observed between DNKv1.x and GFP control cells. Similar results were observed at an early timepoint (day3) as well as at later timepoints (days 14 and 21) (data not shown). These data show that both Kv1.3 channel blockers and DNKv1.x transduction are capable of suppressing the proliferation of CD8^+^ cells and suggest the possibility of an effect on CD8^+^ T cell differentiation.

**Figure 3:**
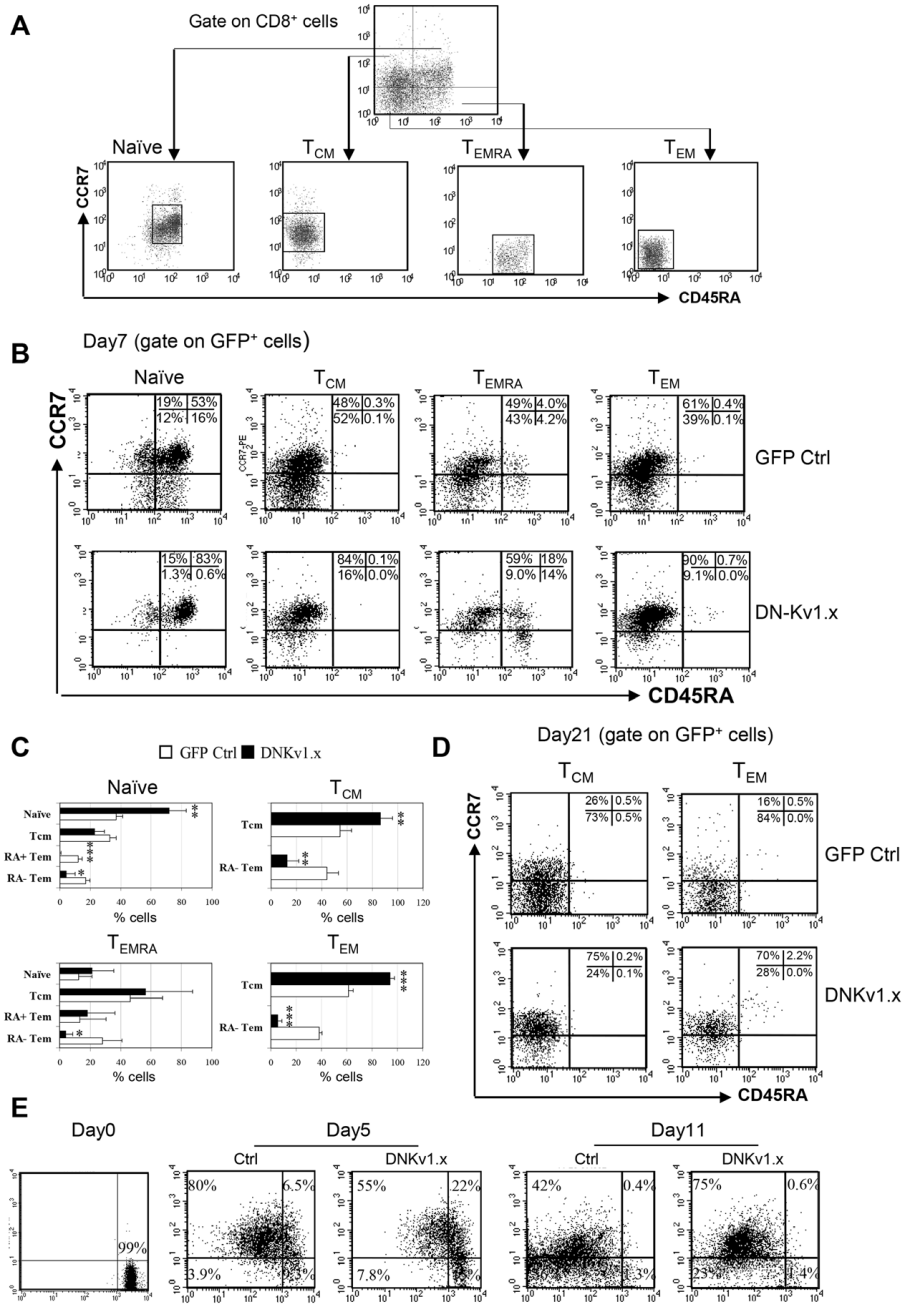
Inhibitory effects of Kv1.3 blockade in the differentiation and homeostatic maintenance of T_EM_ CD8 cells. (A) Naïve, T_CM_, T_EMRA_ and T_EM_ CD8 subpopulations were sorted from CD8+ gated cell population based on surface markers of CCR7 and CD45RA. Sorted individual subsets within the respective gates shown were transduced with a DN-Kv1.x and GFP control. After 7 days of transfection, the percentages of each subset in gated GFP+ CD8+ cells were analyzed by flow cytometry. Gate for expression of GFP was established using untransduced controls. (B) FACS profiles are representative of three separate experiments using cells from different donors. The percentage of cells in each quadrant is indicated. (C) The percentages of CD8 subsets displaying GFP fluorescence from each single transfected subpopulation are presented as mean ± SD of three experiments. Values that are significantly different from that of GFP control are indicated as follows: *, *p*<0.05, **, *p*<0.01; ***, *p*<0.005. (D) Representative FACS profiles of phenotypical changes of transduced T_CM_ and T_EM_ subsets 21 days after transfection. (E). FACS-sorted CCR7- (T_EM_/T_EMRA_) were labeled with PKH26 day (2×10–6 M), followed by stimulation with anti-CD3/CD28 for 24 h and then transduced with a lentiviral vector encoding the dominant-negative Kv1.x and GFP control alone at an MOI of ∼8. PKH26 fluorescence was analyzed by flow cytometry at days 0, 5 and 11. FACS plots shown are representative data from two experiments.

We next assessed whether blockade of Kv1.3 affected the differentiation of naïve CD8^+^ T cells into T_EM_, naïve, T_CM_, T_EMRA_ and T_EM._ For these studies, CD8^+^ cells were FACS sorted based on their surface expression of CD8, CCR7 and CD45RA (Fig. 3A), transduced with DN-Kv1.x or GFP control, incubated for seven days, then analyzed for their differentiation state. Similar to the effect on unfractionated CD8^+^ cells (Fig. 2C), blockade of Kv1.3 inhibited the differentiation of the individual subsets of T cells into T_EM_ and/or T_EMRA_ subsets (Fig. 3B and C). Thus, the inactivation of Kv1.3 channel function can impair T_EM_ differentiation and proliferation. Further, transduction of T_EM_ cells with DNKv1.x resulted in the conversion of nearly all (>90%) the CCR7^−^ T_EM_ cells to a CCR7^hi^ T_CM_ phenotype. Importantly, this reversion of T_EM_ into T_CM_ was stable for at least the three weeks’ assay time. In contrast, the majority of CCR7^−^ T_EM_ CD8 cells transduced with the GFP control retained a classical T_EM_ phenotype after 3 weeks (Fig. 3D). To further confirm that the CCR7^+^ cells did revert from CCR7^−^ T_EM_/T_EMRA_ cells in DNKv1. transduced CD8 cells, we used PKH26 staining to monitor CCR7 expression as a function of cellular division. FACS-sorted CCR7^−^ (T_EM_/T_EMRA_) were labeled with PKH26 and analyzed five and eleven days after transduction for PKH26 dilution and CCR7 expression within the GFP^+^ cells (Fig. 3E). While both control and Kv1.3 blocked cells initially acquired equivalent levels of CCR7 expression, levels over time decreased in the control cells but not in DNKv1.x cells, which suggested a sustained reversion of CCR7^−^ T_EM_ into CCR7^+^ cells.

### K_v_1.3 Blockade Suppresses Signal 1-mediated GrB Production in CD8+ T cells

**Figure 4:**
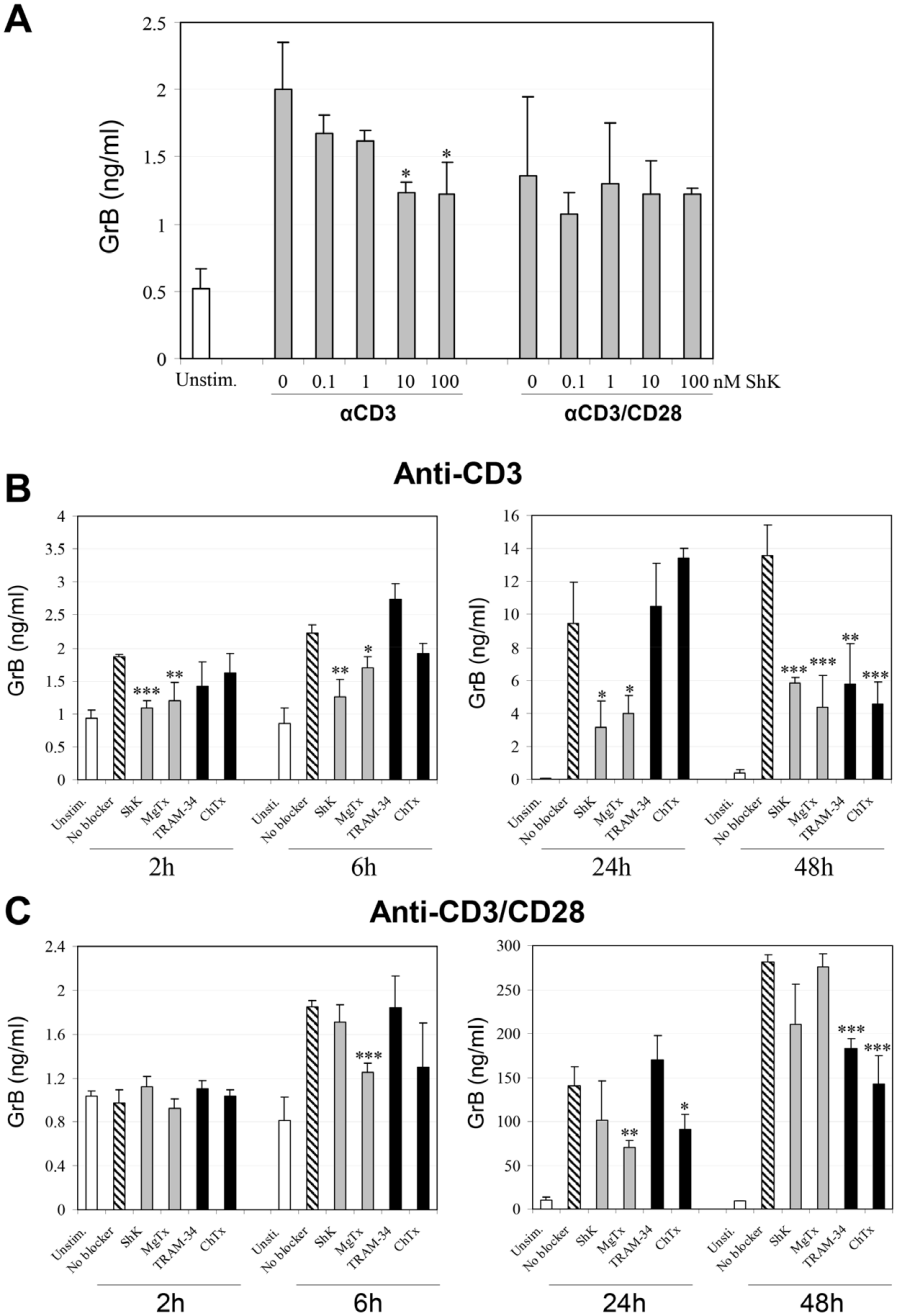
K+ channel blockers inhibit GrB production by activated CD8+ T cells. Freshly isolated CD8+ T cells were pretreated with a Kv1.3 channel blocker, ShK at various concentrations (A) or with ShK (10 nM), MgTX (30 nM), ChTX (50 nM) and TRAM-34 (500 nM) (B) for 3 h, followed by stimulation with anti-CD3/CD28 or anti-CD3 alone. The levels of GrB were measured in cell supernatants by ELISA at 6 h (A) and indicated times (B and C). Data are mean of triplicate ± SD of one representative of three independent and reproducible experiments. Values that are significantly different from that of non-blocker vehicle treated control are indicated as follows: *, *p*<0.05; **, *p*<0.01.

A hallmark of the CD8^+^ T cell activation is the production of cytotoxic proteins such as GrB, which is critically important in the induction of efficient cytotoxicity. Because T_EM_/T_EMRA_ subsets represent a greater proportion of the GrB-secreting pool among CD8^+^ T cells (Fig. S1 and Fig. S2), we reasoned that the impaired T_EM_ differentiation and/or a reversion of T_EM_ into T_CM_ might be associated with a downregulation of GrB expression in CD8^+^ T cells in which Kv1.3 signaling was blocked. As Fig. 4A demonstrates, treatment of purified CD8^+^ T cells with ShK, a selective Kv1.3 blocker, significantly inhibited anti-CD3 induced GrB release in a dose-dependent manner, while the ability of ShK to suppress GrB release by CD8^+^ T cells in response to a more potent stimulus, anti-CD3/CD28, was not significant.

To determine the specific role of the Kv1.3 channel in mediating GrB release, we next assessed the effects of several different channel blockers on GrB release by CD8^+^ T cells at various time points. K_v_1.3 blockade was compared with two other ion channel blockers: ChTx and TRAM-34, which are specific blockers of KCa3.1, a Ca^2+^-dependent potassium channel that dominates in activated naïve and T_CM_ subsets. MgTx and ChTx were used at 30 nM and 50 nM, respectively which corresponded to IC_50_ values in anti-CD3-induced CD8^+^ T cell proliferation (date not shown). TRAM-34 was used at 500 nM based on the IC_50_ in anti-CD3-induced T cell proliferation [30]. As shown in Fig. 4B, treatment of CD8^+^ T cells with either of the two K_v_1.3 blockers, ShK and MgTx, resulted in significant inhibition of anti-CD3 induced GrB production. TRAM-34 and ChTx treatment did not affect GrB release within 24 h, but did inhibit GrB production 48 hours after anti-CD3 stimulation. Interestingly, although ShK inhibited anti-CD3-induced GrB release at a comparable level to MgTx, it failed to exhibit the significant inhibition on anti-CD3/CD28-mediated GrB release that was observed with MgTx (Fig. 4C). It should be noted that the inhibitory effects were not likely due to non-specific cytotoxicity by K^+^ channel inhibitors, since cell viability was quite similar between the untreated and treated groups as judged by both trypan blue uptake and 7AAD staining. We also examined the effect of these blockers on perforin production and found that they had no significant effect on perforin production by either unfractioned CD8 cells or CD8 subsets (Fig. S3).

### Kv1.3 Inhibitors do not Affect CD107a Expression on Activated CD8^+^ Cells

**Figure 5:**
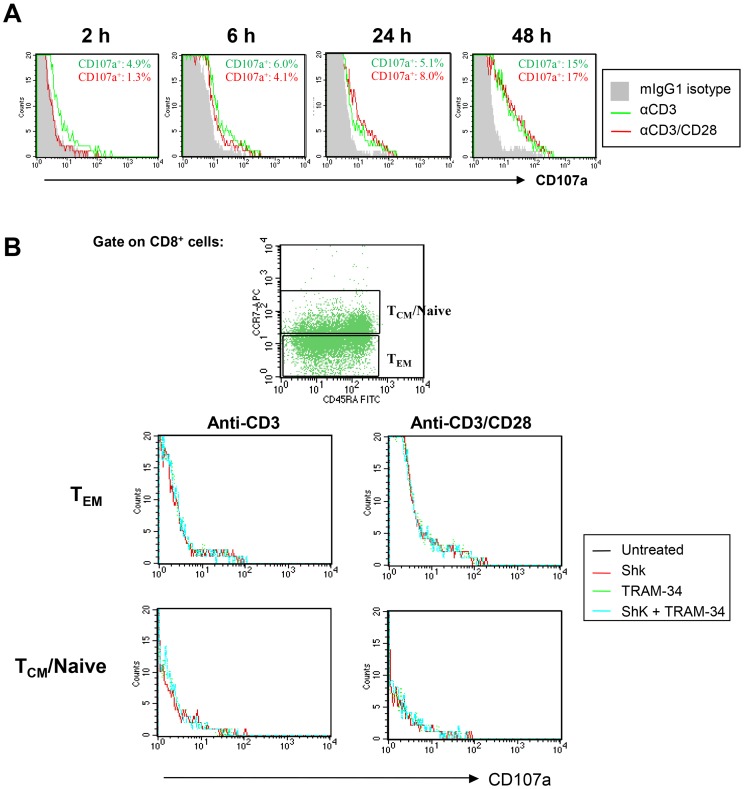
K+ channel blockers do not affect CD107a expression on activated CD8+ T cells. (A) Freshly isolated CD8+ T cells were stimulated with anti-CD3/CD28 or anti-CD3 for 24 hours. Cells were then stained with a CD107a-specific mAb, or an IgG1 isotype control (filled histogram) at the indicated times. (B). CD8+ T cells were pretreated with ShK (10 nM), MgTX (30 nM), ChTX (50 nM) and TRAM-34 (500 nM) for 3 h, followed by stimulation with anti-CD3/CD28 or anti-CD3 alone for 6 hours. Surface expression levels of CD107a were then analyzed by flow cytometry. FACS plots shown are representative data from three separate experiments.

Given the possibility that the reduced GrB production by K^+^ channel blockers in activated CD8^+^ T cells might reflect a diminished capacity of these cells to degranulate, we evaluated the expression levels of CD107a, which is a marker for cytotoxic CD8^+^ T cell degranulation [31]. As shown in Fig. 5A, surface expression of CD107a was induced in CD8^+^ cells beginning two hours after stimulation with anti-CD3/CD28 or anti-CD3 and increased thereafter, indicating that degranulation had occurred in activated CD8^+^ T cells. Analysis of the functional consequences of K^+^ blockade on degranulation revealed that the frequency of CD107a^+^ cells presented in untreated CD8^+^ T cells was comparable to that of those treated with K^+^ channel blockers.

### Kv1.3 Blockade Ameliorates GrB-mediated Toxicity to Neural Cells

**Figure 6:**
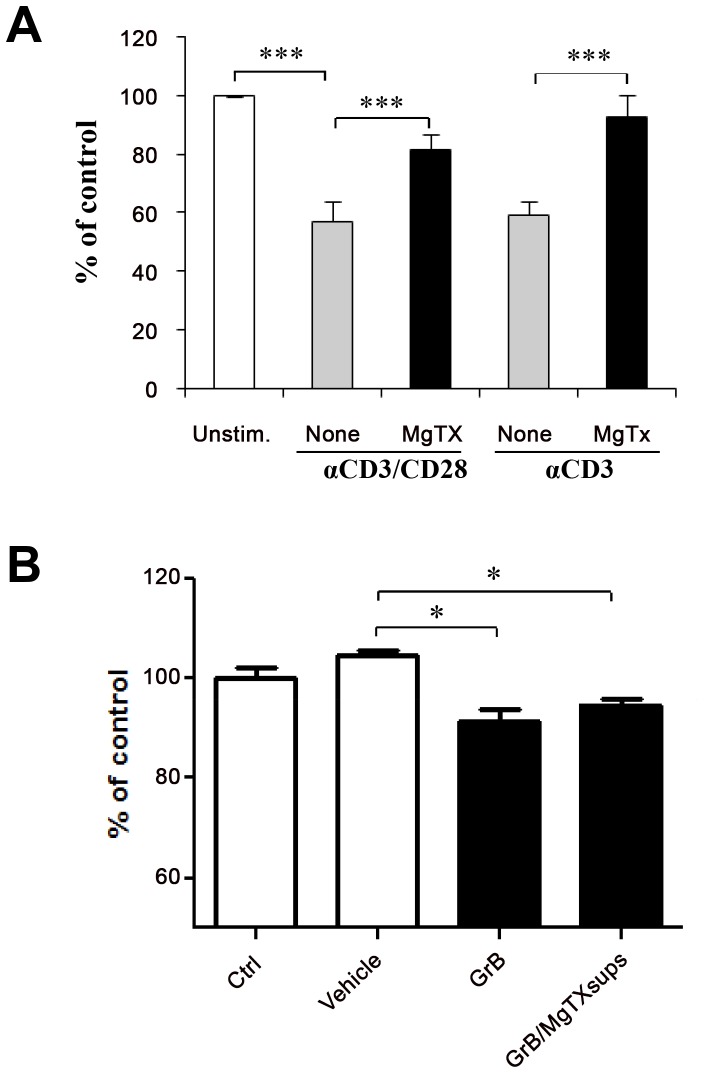
Kv1.3 channel blocker attenuates GrB mediated neural cell toxicity. Freshly isolated human CD8+ T cells were simulated with anti-CD3 or anti-CD3/CD28 in the presence or absence of MgTx. Cultured supernatants were collected at 3 days after stimulation. (A). Human neural cells cultured on poly-D-lysine pre-coated 96 well plates were pretreated with supernatants from non-activated CD8 T cells (Unstim.), anti-CD3 or anti-CD3/CD28-activated CD8+ T cells without MgTx (none), and with MgTx (MgTx). After 24 hours of treatment, CellQuanti-blue dye was added in each well for 30 minutes. Fluorescence was then detected using a plate reader. Cell viability was quantified by fluorescence intensity. (B). MgTx (30 nM) was added to culture media and incubated for 3 days. Human NPCs were treated with supernatants without MgTx (Ctrl), MgTx contained sups with vehicle treatment, with GrB alone (GrB) or MgTx containing sups plus GrB treatment (GrB/MgTx sups). Neurotoxicity was evaluated by cell viability quantified by fluorescence intensity. The fluorescence intensity in each group is plotted as percent relative to that in non-activated cells (Unstim.) or control cells (Ctrl). Data are mean of triplicate ± SD of one representative of three independent experiments. Values that are significantly different from that of vehicle treated control are indicated as *, *p*<0.05; **, *p*<0.01; ***, *p*<0.005.

To assess the potential functional relevance of Kv1.3 blockade in GrB-mediated neuronal toxicity, we compared the effects of conditioned media from activated CD8^+^ T cells treated with MgTx or vehicle on neural cell viability. The results in Fig. 6A show that cultured supernatants of activated CD8^+^ T cells induced significant reduction in cell viability, as compared with non activated CD8^+^ T cell-derived supernatants. The addition of MgTx to CD8^+^ T cells significantly attenuated activated conditioned T cell supernatant mediated cell toxicity. Immunodepletion of GrB with a specific antibody from activated CD8^+^ T cell-derived supernatants demonstrated that the cell toxicity was specifically induced by GrB (data not shown). To exclude the possible beneficial effects on neuronal survival of residual MgTx in the conditioned media that was added to the human neural cells (unlikely because of its short half life), media containing the same concentration of MgTx was incubated for 72 hours and was used as pretreatment prior to recombinant GrB (Fig. 6B). Pretreatment of neurons with day 3 MgTx-containing supernatants did not block the toxic effects of GrB on neural cell neurogenesis showing that there was no active drug in the 3 day old supernatants transferred into the neuronal cultures.

## Discussion

CD8^+^ T cells have been shown to contribute to autoimmune diseases such as MS. Understanding the mechanisms by which they mediate pathology may help to identify new targets for therapeutic intervention. In the present studies, we confirmed our previous finding that the Kv1.3 channel is highly expressed in the cell membrane of activated CD8^+^ T cells [13], and we now show that Kv1.3 blockade effectively inhibited proliferation of CD8^+^ T cells in response to anti-CD3 alone and, in some cases, in combination with anti-CD28. Our data further indicate that Kv1.3 signaling is important for both differentiation of naïve and central memory into effector memory cells as well as for maintenance of effector status. Transduction of activated CD8^+^ cells with DNKv1.x resulted in a significant reduction in CCR7^−^T_EM_ cells, with a corresponding increase in CCR7^+^ T_CM_ cells. These results indicate a functional role of Kv1.3 channel in the regulation of proliferation as well as differentiation of CD8^+^ T cells, which is important since we previously showed that anti-proliferative drugs alone do not reproduce the conversion to CCR7^+^ T_CM_ cells seen with KvDN transduction.

Effector memory T cells are thought to be the pathogenic subset of T cells in autoimmune diseases. Maintenance of memory cells is likely to be a dynamic process that requires activation of a number of signaling pathways. Our data indicate a role for Kv1.3 in this process for CD8^+^ T cells. Specifically, we show that a significantly higher proportion of DNKv1.x transduced T_EM_ convert to CCR7^+^ T_CM_ cells as compared to GFP-control transduced T_EM_, which is surprising and suggests there is more plasticity along this lineage pathway than previously thought. Although, our data are consistent with a recently described lineage fate mapping model of CTLs in mice, which also found greater than 50% of T_EM_ could revert to T_CM_ [32]. We recently showed that Kv1.3 blockade causes G2 M cell cycle blockade and arrests CD4^+^ T cells as T_CM_ [33]. Taken together, these results indicate a significant role for Kv1.3 in the proliferation and differentiation of both T_EM_ CD4^+^ and CD8^+^ cells.

Tissue destruction in autoimmune disease is likely to be mediated by both CD4^+^ and CD8^+^ effector T cells, through a variety of functions including release of proteases such as GrB. As the majority of CD8^+^ T cells expressing GrB are indeed T_EM_ and T_EMRA_ subpopulations of resting [34], [35] and early (within 24 hours) activated CD8^+^ T cells selectively express high levels of Kv1.3, we hypothesized that there might be a link between Kv1.3 expression and GrB secretion. We assessed the release of GrB after treatment with the Kv1.3 blocker, ShK, and found a significant decrease in GrB expression in activated CD8^+^ T cells, without any significant inhibition of cell proliferation, which is consistent with the profile of a T_EM_ cell. These results provide a further piece of evidence suggesting that therapeutic potential of Kv1.3 channel blockade may mediate its effects through CD8^+^ T cells, as well as CD4^+^ T cells. Notably Kv1.3 blockers at concentrations that inhibit T_EM_ cells should spare other tissues or cells, because lymphocytes are especially susceptible due to their use of homotetrameric Kv1.3 complexes, whereas Kv1.3 in other cell types (e.g. neurons) is typically in heteromeric complexes.

We also found that unlike GrB, perforin was not significantly influenced by Kv1.3 channel blockade. This observation is consistent with reports that perforin release may occur through different molecular pathways and that the toxicity in these cells can be independent of perforin [16] [36], supporting an important role for GrB in neurodegeneration.

Since T cells express two major potassium channels Kv1.3 and KCa3.1, we sought to distinguish the unique role of Kv1.3 in GrB production and identify whether there was an additional role for K_Ca_3.1, another K^+^ channel known to be important in T cells. Thus, we compared the effects of K_v_1.3 channel blockers and K_Ca_3.1 channel blockers on GrB production in CD8^+^ T cells. The K_v_1.3 channel blockers ShK and MgTx both potently inhibited early (24 hours or less) GrB production in anti-CD3-activated CD8^+^ T cells, while less on non-specific blockers that more weakly target Kv1.3 and also K_Ca_3.1 channels blockers did not produce such bban effect. This result suggests that the early decrease in GrB production detected in anti-CD3 stimulated CD8^+^ T cells was specifically mediated by K_v_1.3 channel inhibition on the memory T cells, which are the only subtype of CD8^+^ T cells that can produce GrB in this time frame. This finding is consistent with the early response of T_EM_ cells to an immune challenge in which they initiate immediate effector functions such as cytokine secretion or cytotoxicity [37]. In contrast, there was no inhibition of GrB production in CD8^+^ T cells during the early timepoints when the KC_a_3.1 channel was blocked by TRAM-34 and ChTx. As KC_a_3.1 acts as the functionally dominant K^+^ channel in naïve and T_CM_ subsets, and is not transcriptionally up-regulated until 12 hours after activation [4], this later inhibitory effect of TRAM-34 is most likely due to inhibition of activated naïve and T_CM_ subsets, which are typically not capable of immediate effector functions. Although ChTx inhibits both K_v_1.3 and K_Ca_3.1 channels in human T lymphocytes, its suppression of GrB release by CD8^+^ T cells at late time points after anti-CD3 stimulation suggests its efficient blocking of K_Ca_3.1 channels is predominant, since it had no effect on GrB production at the early timepoints (within 24 h) when K_v_1.3 is significantly upregulated. The potential relevance of these findings is supported by both results from our previous study [16] in which the toxicity of activated CD8 T cell supernatants to cultured human fetal neurons was diminished when GrB was immunodepleted, as well as the present study in which Kv1.3 blockade inhibited GrB in activated cytotoxic T cell supernatants and decreased their toxicity to neural cells. It is worth noting that while the present study provides evidence for the effects of these short half-life Kv1.3 blockers on T cells, we have also previously shown a separate effect on neuronal cell death pathways that are regulated by Kv1.3 [15].

As activation of T cells occurs as a two-signal process, we investigated the role of Kv1.3 blockers in the individual events of the process. Our previous work on CD4^+^ T cells showed that while the Kv1.3 channel blocker, ShK effectively inhibited anti-CD3-induced activation and cytokine release, the addition of a potent costimulatory signal, anti-CD28 overcame the blockade [27]. Our present observations in which ShK suppressed early anti-CD3-induced GrB release in CD8^+^ T cells, but failed to inhibit CD3/CD28-induced GrB release suggest that a similar mechanism could occur in CD8^+^ T cells as well. However, unlike ShK, MgTx potently inhibited GrB production in CD8^+^ T cells induced by anti-CD3/CD28 as well as anti-CD3 alone. Because MgTx is chemically distinct from ShK, the differential effects of these selective Kv1.3 blockers may imply that MgTx operates through a different mechanism which bypasses the CD28-mediated signal, although it may also relate to increased potency or stability of this compound in vitro.

Further studies are required to elucidate the mechanism(s) by which K^+^ channel blockers down-regulate GrB secretion in CD8^+^ T cells. While our results showed that K^+^ channel blockers do not diminish a marker of degranulation, CD107, we did not exclude either the possibility of a defect in the GrB production, or a later stage of exocytosis that is independent of LAMP-1 detection in the plasma membrane. The former mechanism, that empty granules (not containing GrB) can be still detected by CD107a, has been documented for CD8^+^ T cells that lack cytotoxic effector functions [38]. This may suggest that the Kv1.3 inhibitory function directly targets granule production or even GrB gene gene expression via effects on transcription factors, such as AP-1, which is known to be required for induction of GrB expression by antigenic stimulation [39], [40], [41]. An alternative explanation for our data is the possibility is that K^+^ channel blockers affect the mobilization of intracellular free calcium ([Ca^2+^]i), thereby effecting later stages of exocytosis [42], [43]. We thus hypothesize that Kv1.3 blockade, which inhibits intracellular accumulation of calcium in CTL, may block vesicle exocytosis, but more extensive research is required to clarify how Kv1.3 blockade may specifically target this pathway.

Our study has several limitations. As mentioned at the outset, the autoantigen in MS is unknown and may be varied so we could not demonstrate specificity of our findings to myelin-reactive T cells, nor do we argue that such is the case. MBP reactive T cells can be isolated but their precursor frequency is so low as to make the detailed dosing and transduction experiments, described herein, impossible. The pharmacological blockers are short-acting and do not have sustained effects in longer in vitro assays requiring use of the Kv1.x lentivirus system. While this provided robust confirmation of our pharmacological data, it is arguably different than blocking the channel pore with a small molecule inhibitor and our data should be interpreted in that light. Finally, lentiviral transduction seems to obviate our ability to measure intracellular GrB thereby preventing us from asking critical questions related to whether Kv1.3 blockade inhibits production of GrB granules or rather just has an effect on secretion. Nonetheless, the data presented herein significantly extend our prior report on CD8 T cells, which was limited to physiological proof of Kv1.3 channels on CD8 T cells and effects on proliferation, but did not examine differentiation, reversion to Tcm, or GrB release and neurotoxicity.

In conclusion, this study demonstrates that Kv1.3 is a component of the pathways that regulate GrB production in activated CD8^+^ T cells. While an extensive literature has documented the role of Kv1.3 in CD4^+^ T cell direct effector function in autoimmune diseases, the novel additional inhibitory effects described herein on GrB release from CD8^+^ effector cells may be especially relevant in MS, as a means of protecting neurons and axons from direct cytoxicity [15], [16].

## Supporting Information

Figure S1 **CD8+ T cells produce GrB in response to anti-CD3/CD28 or anti-CD3.** (A) Purified CD8+ T cells were stimulated with anti-CD3/CD28 or anti-CD3 for 24 h (A) or in a time course (B) Then, cell-free supernatants were collected and assayed by ELISA for GrB secretion. Data are mean of triplicate ± SD of one representative of three independent and reproducible experiments. The value was significantly different from non-stimulated control. (**, *p*<0.01; ***, *p*<0.005) (C) Flow cytometric analysis of intracellular GrB in activated CD8+ T cells. Anti-CD3/CD28 (red line) or anti-CD3 (green line) stimulated CD8+ T cells were stained with a GrB-specific mAb, compared with an IgG1 isotype control (filled histogram). This figure is representative of three different donors. (D) Purified CD8+ T cells were stimulated with anti-CD3/CD28 for 24 h. Cells were then immunostained for GrB (red) in combination with CD8 (green) and subsequently viewed by immunofluorescence microscopy. Isotype control failed to show any specific staining. Original magnification, ×100. Image is representative of three different donors.
(PPT)

Figure S2 **Differential expression of GrB in CD8+ T cell subsets.** (A) Differential gating on freshly isolated CD8+ T cells based on CCR7 and CD45RA expression revealed four populations (Naïve, T_CM_, T_EM_ and T_EMRA_) with distinct patterns of GrB expression. (B) kinetic changes of GrB expression in CD8+ T cells following stimulation with anti-CD3 alone or anti-CD3/CD28. FACS profiles are representative of three different donors.
(PPT)

Figure S3 **K+ channel blockers do not affect perforin production by activated CD8+ T cells.** (A) Freshly isolated CD8+ T cells and (B) FACS sorted naïve, T_CM_, T_EM_ (CCR7-CD45RA-) and T_EMRA_ (CCR7-CD45RA+) (B) were pretreated with a Kv1.3 channel blocker, ShK at various concentrations and at 10 nM, respectively. 3 hr after treatment, cells were stimulated with anti-CD3/CD28 or anti-CD3 (A) and anti-CD3 alone (B). The levels of perforin were measured in cell supernatants by ELISA at 24 h.
(PPT)
