# Supplementary figures and images for: Blockade of Kv1.3 Potassium Channels Inhibits Differentiation and Granzyme B Secretion of Human CD8+ T Effector Memory Lymphocytes
Source: PLoS One. 2013 Jan 30;8(1):e54267. doi: 10.1371/journal.pone.0054267 (PMC3559683; doi:10.1371/journal.pone.0054267)

## Slide 1
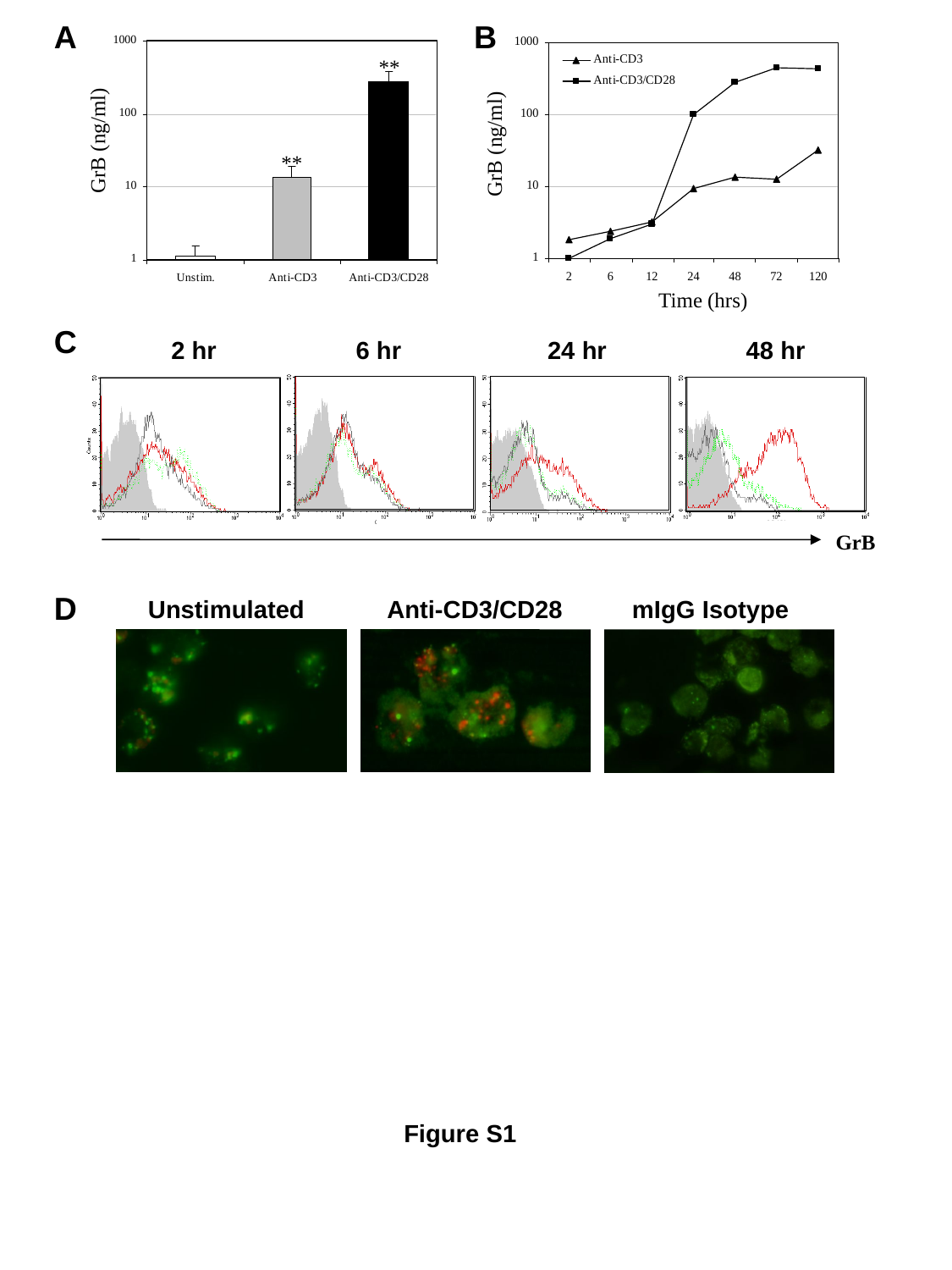

A B
C
D
**
GrB (ng/ml)
GrB (ng/ml)
**
Time (hrs)
2 hr 6 hr 24 hr 48 hr
GrB
 Unstimulated Anti-CD3/CD28 mIgG Isotype
Figure S1

Supplement: Figure S1 — CD8+ T cells produce GrB in response to anti-CD3/CD28 or anti-CD3. (A) Purified CD8+ T cells were stimulated with anti-CD3/CD28 or anti-CD3 for 24 h (A) or in a time course (B) Then, cell-free supernatants were collected and assayed by ELISA for GrB secretion. Data are mean of triplicate ± SD of one representative of three independent and reproducible experiments. The value was significantly different from non-stimulated control. (**, p<0.01; ***, p<0.005) (C) Flow cytometric analysis of intracellular GrB in activated CD8+ T cells. Anti-CD3/CD28 (red line) or anti-CD3 (green line) stimulated CD8+ T cells were stained with a GrB-specific mAb, compared with an IgG1 isotype control (filled histogram). This figure is representative of three different donors. (D) Purified CD8+ T cells were stimulated with anti-CD3/CD28 for 24 h. Cells were then immunostained for GrB (red) in combination with CD8 (green) and subsequently viewed by immunofluorescence microscopy. Isotype control failed to show any specific staining. Original magnification, ×100. Image is representative of three different donors. (PPT) [file pone.0054267.s001.ppt]

## Slide 1
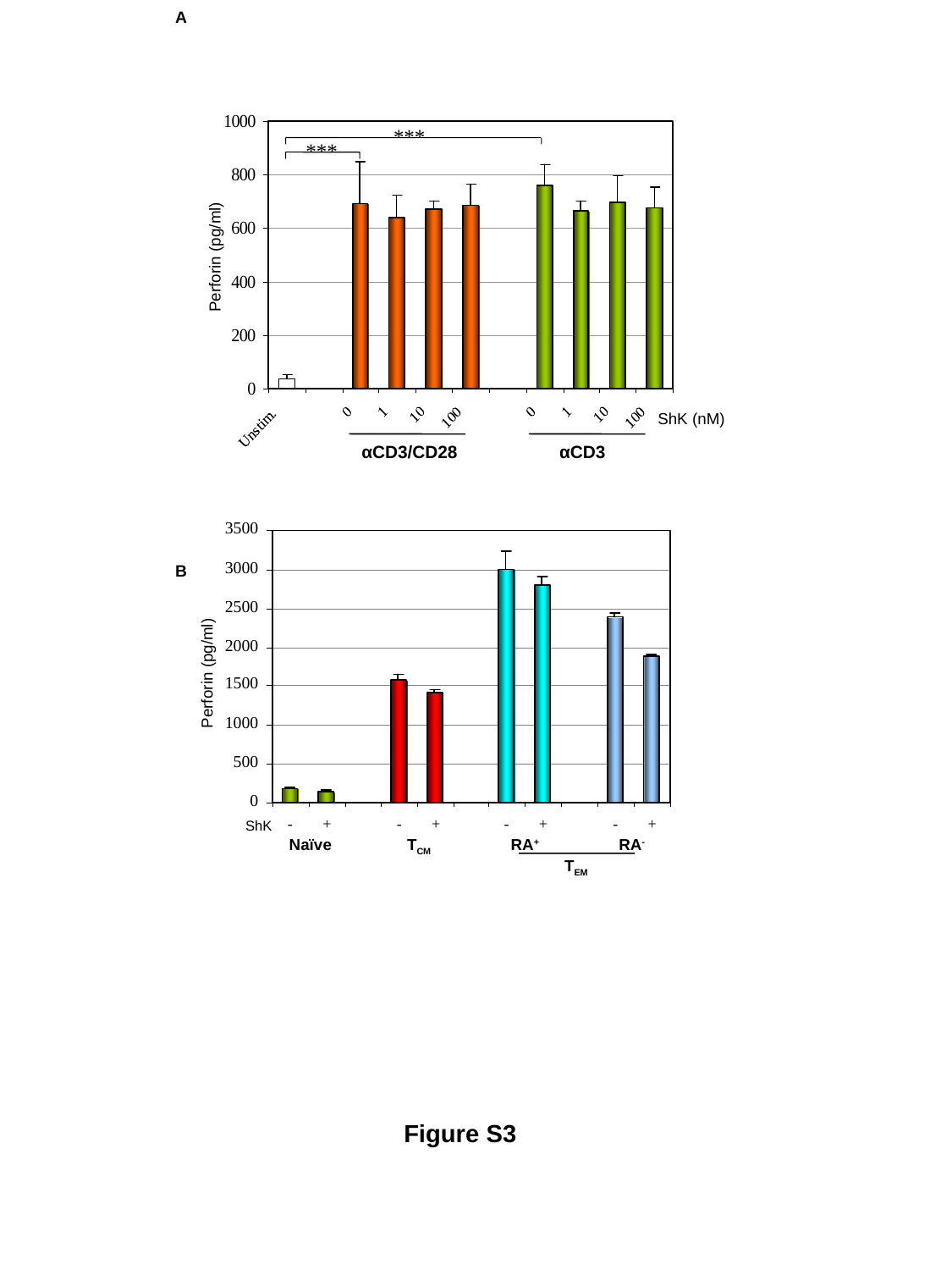

A
B
***
***
Perforin (pg/ml)
ShK (nM)
αCD3/CD28 αCD3
Perforin (pg/ml)
ShK
Naïve TCM RA+ RA-
 TEM
Figure S3

Supplement: Figure S3 — K+ channel blockers do not affect perforin production by activated CD8+ T cells. (A) Freshly isolated CD8+ T cells and (B) FACS sorted naïve, TCM, TEM (CCR7-CD45RA-) and TEMRA (CCR7-CD45RA+) (B) were pretreated with a Kv1.3 channel blocker, ShK at various concentrations and at 10 nM, respectively. 3 hr after treatment, cells were stimulated with anti-CD3/CD28 or anti-CD3 (A) and anti-CD3 alone (B). The levels of perforin were measured in cell supernatants by ELISA at 24 h. (PPT) [file pone.0054267.s003.ppt]
