# Supplementary material for: Blockade of Kv1.3 Potassium Channels Inhibits Differentiation and Granzyme B Secretion of Human CD8+ T Effector Memory Lymphocytes
Source: PLoS One. 2013 Jan 30;8(1):e54267. doi: 10.1371/journal.pone.0054267 (PMC3559683; doi:10.1371/journal.pone.0054267)
Supplement: Figure S2 — Differential expression of GrB in CD8+ T cell subsets. (A) Differential gating on freshly isolated CD8+ T cells based on CCR7 and CD45RA expression revealed four populations (Naïve, TCM, TEM and TEMRA) with distinct patterns of GrB expression. (B) kinetic changes of GrB expression in CD8+ T cells following stimulation with anti-CD3 alone or anti-CD3/CD28. FACS profiles are representative of three different donors. (PPT) [file pone.0054267.s002.ppt]

## Slide 1
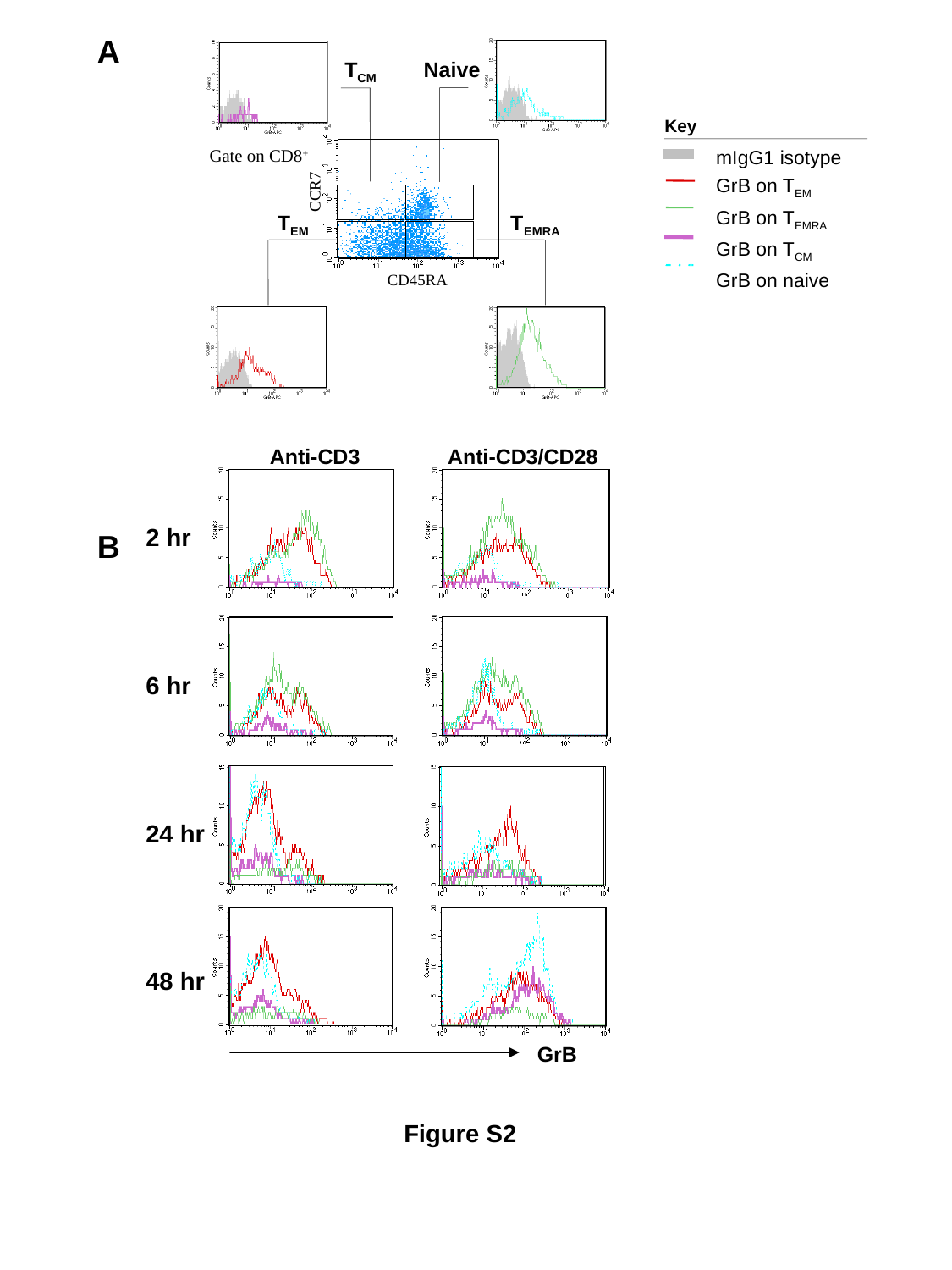

A
B
TCM Naive
Key
mIgG1 isotype
GrB on TEM
GrB on TEMRA
GrB on TCM
GrB on naive
Gate on CD8+
CCR7
TEM TEMRA
CD45RA
Anti-CD3 Anti-CD3/CD28
2 hr
6 hr
24 hr
48 hr
GrB
Figure S2
